# “The Idea of Not Having to Prove Anything in the Way I Needed, That Would Be Really Helpful”: Exploring Adolescents’ and Parents’ Understanding of Youth Participation During Patient-Initiated Brief Admission Enrollment in Psychiatric Inpatient Care

**DOI:** 10.1177/10497323251340935

**Published:** 2025-06-07

**Authors:** Jennie Moberg

**Affiliations:** 1 Department of Social Work, 376168 Stockholm University, Stockholm, Sweden

**Keywords:** adolescents, ecological systems theory, epistemic injustice, family-oriented work, mental health recovery, inpatient care, participation, patient-initiated brief admission (PIBA)

## Abstract

Enrollment in psychiatric inpatient care represents a pivotal moment in a young person’s life, one that often reverberates through the entire family. This study seeks to illuminate how adolescents and parents navigate youth participation during Patient-Initiated Brief Admission enrollment, shedding light on how such involvement fosters recovery from mental health problems. Drawing on interviews with five adolescents and five parents, the study unpacks the intricate dynamics of participation, with thematic analysis revealing the underlying patterns and complexities. To deepen the understanding, theoretical frameworks of epistemic injustice and ecological systems theory are employed, offering rich perspectives on the interplay of knowledge, power, and the parental role in shaping participatory experiences. The findings show that participation is not a static concept but a relational process, rooted in trust and profoundly influenced by adolescents’ ability to articulate their care needs. This capacity emerges as a cornerstone for enhancing meaningful participation and promoting recovery. Moreover, including parents in the enrollment process provides a dual vantage point—highlighting both the benefits and the challenges of inpatient care—while simultaneously enriching the participatory experience. The study underscores that youth participation in inpatient care is inherently dynamic, evolving through relationships with adults and fluctuating in intensity and frequency. Recognizing adolescents as emerging adults, capable of increasing autonomy despite health challenges, is essential to fostering equitable and empowering care. This perspective, crucial during inpatient enrollment, serves as a safeguard against epistemic injustice, ensuring that participation remains a pathway to recovery rather than a barrier.

## Introduction

Enrolling in psychiatric inpatient care marks a pivotal moment in an adolescent’s^1^ life. Stanton et al. (2017) emphasize the vulnerability of young people during both admission and discharge, implicitly suggesting that these experiences can have significant and lasting consequences. However, despite the profound impact of such experiences, young people in need of mental health care may face barriers to having their perspectives legitimized by adults such as parents and professionals (Hansen et al., 2021; cf. Llewellyn-Beardsley et al., 2022). Moreover, as vulnerable adolescents rely on adults to balance participation with protection, this reliance can inadvertently limit their agency when they encounter mental health care (Bjønness et al., 2024). Knowledge hierarchies within the inpatient care setting and questions about credibility may further restrict their involvement, potentially hindering the full realization of participation as outlined in the Convention on the Rights of the Child (Greenblatt et al., 2024; UNCRC, 1989; Yamaguchi et al., 2023).

Article 12 of the UNCRC (1989) emphasizes the importance of including young people’s voices in various forms of decisions. This points to meaningful participation, especially for adolescents with disabilities or complex mental health problems, who may face a “double denial” of rights—first due to their age, then their perceived lack of competence (Lundy, 2007, p. 935). Consequently, tensions might arise during enrollment in inpatient care due to competing interpretations among adolescents, parents, and professionals, increasing the risk of young people not being valued as “knowers” (cf. Fricker, 2007; Greenblatt et al., 2024; Klyve, 2019). Drawing on this, balancing young people’s right to participate with the duty to protect them is a challenge among staff and parents, as highlighted in several studies (Bjønness et al., 2020; Moberg, 2024; Stanton et al., 2020; Viksveen et al., 2024). Accordingly, and to support youth recovery, there is a need for efforts that move beyond tokenistic participation (where young people are present but lack real influence, Shier, 2001) toward strengthening genuine involvement (Coyne & Harder, 2011; Daly, 2018; Lundy, 2007).

While research on youth mental health recovery expands, the factors that young people identify as essential to their personal recovery require further exploration (Kelly & Coughlan, 2019; Rayner et al., 2018). In this study, recovery is framed as integrating knowledge, power, and participation. This conceptualization extends beyond symptom reduction, recognizing recovery as a personal and multifaceted process (Leamy et al., 2011; Moberg et al., 2022). Recovery-oriented work with young people embraces a holistic approach, emphasizing their unique abilities, personal goals, and individual strengths (Hughes et al., 2016; Naughton et al., 2020; Rayner et al., 2018). Additionally, supporting personal recovery often involves committed parents who help guide and sustain the process (Hassett et al., 2018; Hopkins et al., 2021; Kelly & Coughlan, 2019; Naughton et al., 2020; Reid et al., 2024). This orientation aligns with the CHIME framework (Connectedness, Hope, Identity, Meaning, and Empowerment; Leamy et al., 2011; cf. Ballesteros-Urpi et al., 2019; Schneidtinger & Haslinger-Baumann, 2019), which emphasizes the importance of connectedness in recovery. For adolescents in mental health care, this connectedness is often rooted in relationships with parents and staff (Kelly & Coughlan, 2019). Building on this foundation, this study frames participation as a *relational* and *dynamic* process (Cahill & Dadvand, 2018), advocating a shift from passive information reception to active collaboration (cf. Bjønness et al., 2020). By positioning participation as a core element of youth recovery (Moberg, 2024; Moberg et al., 2022), the study underscores its role in fostering empowerment and meaningful engagement within mental health care systems.

In Swedish Child and Adolescent Psychiatric Care (CAP), family-oriented collaboration is emphasized (Bäckström et al., 2024; Linderborg et al., 2024; cf. Bronfenbrenner, 1979). However, parents’ perceptions of their child’s needs may differ from the young person’s views (Davies & Wright, 2008; cf. Holmqvist et al., 2023). Therefore, it has been described as essential to consider the systemic influence of parents on youth participation during inpatient care (Bjønness et al., 2022; Law et al., 2020; Moberg, 2024). This stance mirrors broader trends toward person-centered models of care (Eckerström et al., 2019; McCormack et al., 2011), and Patient-Initiated Brief Admission (PIBA) in CAP exemplifies such a model. PIBA, initially for adults, allows adolescents between the ages of 13 and 17 to self-initiate short-term (1–3 days) psychiatric inpatient care during crises (Moberg, 2024; Moberg & Schön, 2022). It empowers young people by enabling access based on their subjective judgment and a self-formulated contract with parents and staff, bypassing emergency visits. This approach has the potential to enhance youth participation while illustrating the shift toward recovery-oriented mental health care that prioritizes empowerment and a holistic view. Recent studies have explored how PIBA affects participation and care outcomes. Värnå et al. (2025) conducted a systematic review highlighting patient experiences with PIBA in psychiatric care, emphasizing its role in enhancing participation and recovery. Additionally, Eckerström et al. (2024) examined the effects of PIBA on psychiatric care consumption, demonstrating its potential to reduce emergency admissions and improve care continuity. However, given its recent introduction in Swedish CAP, studies on its outcomes for adolescents and parents remain limited. Early findings suggest a reduction in emergency interventions and effects on families (Johansson et al., 2023; Lantto et al., 2023; cf. Hultsjö et al., 2023). Lindkvist et al. (2022) found that PIBA may bolster adolescents’ confidence in assessing their mental health needs, though staff attitudes significantly influence care experiences. Notably, recent studies reveal differing staff perspectives on youth participation and their concerns about parental dominance in PIBA decision-making (Moberg, 2024; Moberg & Schön, 2022).

In conclusion, implementing PIBA challenges traditional inpatient models that typically position staff and parents as the primary experts (cf. Accurso et al., 2020). Moreover, by enabling young people to more independently decide on inpatient care enrollment, PIBA highlights shifts in knowledge among stakeholders, warranting further scrutiny. Therefore, this study aims to explore adolescents’ and parents’ attitudes toward youth participation during PIBA enrollment in psychiatric inpatient care, and in what way participation may contribute to recovery from mental health problems.

## Theoretical frameworks

The theoretical frameworks underpinning this study address knowledge and power, as well as how young persons’ ecological surroundings impact youth participation and opportunities for recovery. Through the lens of epistemic injustice (Fricker, 2007), it seeks to describe the ways in which individuals suffer injustice because of their status as knowledge carriers and providers. According to Fricker, epistemic injustice deprives people of their fundamental right to know about and keep them from having an equal voice in the creation and exchange of information. It is argued that certain groups are more likely to encounter epistemic injustice, such as minors (Carel & Györffy, 2014) and people with mental health problems (Newbigging & Ridley, 2018). By adopting this theoretical standpoint, it helps to demonstrate how adolescents’ credibility and epistemic position risks being compromised by identity stereotypes and structural marginalization during enrollment in inpatient care (cf. Greenblatt et al., 2024; Klyve, 2019).

As CAP is characterized as a family-oriented practice, we need to better understand how participation develops among adolescents in inpatient care, and how it can foster recovery. Accordingly, we must take into account both the immediate and wider surroundings of adolescents (Foster et al., 2016; Moberg, 2024). Drawing on Bronfenbrenner’s ecological systems theory (1979), this framework highlights how adolescents’ participation in inpatient care is shaped by interconnected systems—micro-, meso-, exo-, macro-, and chronosystems. At the core, the microsystem focuses on immediate interactions between adolescents, parents, peers, and care staff, where dynamics of power and autonomy play a pivotal role (Rayner et al., 2018). The mesosystem expands this by examining connections between microsystems, such as parent–staff communication, which can either enhance or hinder youth participation (cf. Brown et al., 2021). The exosystem covers institutional policies and staff training that influence adolescent participation (Walker & Reibel, 2013). However, when institutional policies rely on rigid protocols or insufficient training, they may inadvertently limit youth agency (Eriksson et al., 2018; McDonald-Harker et al., 2021). This limitation connects to the macrosystem, which reflects cultural norms and power dynamics that shape societal views on adolescent autonomy (Harris & Holman Jones, 2024; Kemp et al., 2016). These cultural influences often inform institutional practices, highlighting the need to critically examine how systemic structures impact participation. Similarly, the chronosystem provides a valuable lens for understanding how youth participation evolves over time. Building on the insights by Kemp et al. (2016), this study considers how personal experiences and systemic changes interact dynamically, emphasizing the importance of adapting approaches to participation as circumstances shift (Bronfenbrenner, 1979).

By combining these theoretical lenses, they generate a deeper understanding of how youth participation is constructed from within, and challenged between different systems when inpatient care is needed.

## Method

### Design and Setting

A qualitative design was chosen for its ability to explore the complexities of human experiences and emotions (Kvale, 1997), as well as topics not yet fully understood (Pyo et al., 2023). This approach is rooted in the assumption that knowledge and reality are subjective and socially constructed (Rose & Kalathil, 2019). Consequently, this study views participation and recovery as multiple subjective truths rather than a single objective reality. The epistemological stance underpinning this research highlights the co-creation of knowledge through researcher–participant interactions. This perspective emphasizes the need for critical reflection on the researcher’s positionality and influence on the research process (Shaw & Gould, 2011; Uggerhøj, 2011).

To deepen our understanding of participation and mental health recovery from a user perspective semi-structured interviews with unrelated adolescents and parents were conducted. These interviews explored PIBA as an intervention potentially enhancing youth participation, while also investigating broader experiences of inpatient psychiatric care. Initially, the study aimed to explore practical experiences of PIBA in inpatient care alone. However, slow implementation of PIBA in CAP made it challenging to recruit individuals with PIBA contracts, affecting the study’s initial focus. To address this, the inclusion criteria were broadened to encompass all inpatient care experiences, allowing participants to reflect on differences between traditional admissions and PIBA principles. This methodological adjustment introduced a significant limitation: many discussions about PIBA were hypothetical rather than based on direct user experience. Nonetheless, participants with firsthand PIBA experience (two out of 10) provided valuable insights into how participatory approaches influence the admission process. These accounts offered a richer understanding of PIBA in practice, highlighting both its benefits and challenges. By combining these insights with reflections from participants familiar only with traditional care models, the study captures a more comprehensive view of adolescent participation in psychiatric inpatient admissions within CAP.

Within the scope of this study, CAP consists of 13 local outpatient units, eight specialized units, an emergency unit, and three inpatient units, with ongoing PIBA implementation in four outpatient units and one inpatient unit, involving about 130 employees (Moberg, 2024). Staff training for PIBA includes a web-based course, with each unit designating a nurse/staff member to oversee implementation. While outpatient services make up most of CAP’s care, inpatient accessibility remains challenging due to high demand exceeding capacity. Inpatient admissions are mostly voluntary and short-term, often lasting a few nights for acute events (CAP, 2023), though longer-term care may occasionally be required.

### Participants and Data Collection

The inclusion criteria for the study required young people to have been admitted to inpatient care within the past two years and to be between 13 and 17 years old at the time. Consequently, adolescents aged 15–19 were approached, along with parents who had experienced inpatient care with their child during this period. The reasons for inpatient care included self-harm, emotional instability, anxiety, manic episodes, suicidal thoughts, eating disorders, compulsive behaviors, and post-traumatic stress. Recruitment of adolescents took place between February 2022 and November 2023, while parent recruitment occurred from October to November 2023. Participants were recruited using purposive and convenience sampling (Etikan et al., 2016), targeting those with specific experiences in CAP inpatient care. Recruitment materials were distributed in CAP outpatient waiting rooms, digital platforms (e.g., The Swedish Partnership for Mental Health, Self-Harm and Eating Disorder Organisation, OCD Association of Stockholm County, and LinkedIn), and via the Stockholm Mental Health Action Network. Interested participants contacted the author via email or text and were later followed up with further information. If interest still remained, interviews were scheduled at the participants’ preferred locations.

A total of 10 qualitative semi-structured interviews were conducted with five adolescents and five parents, all of whom had experience with CAP in three Swedish cities. The final number of participants was determined based on data saturation (cf. Glaser & Strauss, 1967; Hennink & Kaiser, 2017, 2022; Malterud et al., 2016), which was reached when no new themes or insights emerged from the interviews. This approach ensured that the sample size was sufficient to capture the diversity and depth of participants’ experiences relevant to the study’s aim. The youth interviews were conducted in comfortable, private locations, lasting between 50 and 122 minutes (mean length 98 minutes), while parent interviews took place via Zoom and Google Meet, lasting between 64 and 83 minutes (mean length 79 minutes). All interviews were recorded, and written and verbal consent were obtained from all respondents. In-person interviews included a coffee invitation, and participants were given two cinema tickets as a token of appreciation. An interview guide,^2^ developed with input from two senior researchers, included three main questions: (1) How do young people and parents describe experiences of youth participation in inpatient care? (2) What significance do they believe participation has in recovery? (3) How could PIBA foster participation and recovery? The questions were adapted during interviews to ensure flexibility and encourage exploration of emerging themes. Since PIBA was unfamiliar to some respondents, it was explained as a crisis intervention that allows adolescents with a PIBA contract to self-initiate a short admission for relief, without control from parents or staff in CAP.

### Analysis and Rigor

The analysis in this study follows thematic analysis (Braun & Clarke, 2006, 2019), chosen for its flexibility and ability to explore participants’ perspectives and experiences in depth. This method allows for identifying patterns and themes within qualitative data, which is essential for understanding the complex views of young people, parents, and staff. Thematic analysis also supports reflexive engagement with the data, capturing both explicit and implicit meanings, aligning with the study’s aim to explore participation in psychiatric inpatient care.

The data were analyzed in six phases. First, transcripts were read multiple times to familiarize with the material, and initial ideas were noted. With 213 pages of interview data, efforts were made to ensure that potentially relevant content was not overlooked and initial ideas were noted. During this process, sections that appeared unrelated to the research question were carefully reviewed to determine whether they offered insights that could contribute to the analysis. Excluded sections generally focused on topics beyond the study’s scope, such as participants’ general reflections on mental health care or unrelated personal experiences. However, reflexivity remained central throughout the process, and I remained open to unexpected findings that could enrich the understanding of the research question. Following the initial reading of the dataset, the coding process focused on identifying key content specifically related to experiences of participation during PIBA enrollment in psychiatric inpatient care. Next, codes were grouped into themes and sub-themes, which were then reviewed and refined to ensure coherence and relevance. Themes were then clearly defined and named, capturing core meanings related to the research question. Finally, the analysis was written up, supported by illustrative quotes. The concepts of epistemic injustice (Fricker, 2007) and ecological systems theory (Bronfenbrenner, 1979) were applied retrospectively, allowing findings to surface inductively. These theories were then integrated to deepen the analysis and contextualize results.

When interpreting the data, key aspects included self-reflexivity (Berger, 2015) and addressing potential biases to maintain openness. This became particularly important since I have previously worked as a user involvement coordinator in CAP, which challenges my ability to remain as objective and impartial as possible during the analysis process. To strengthen the interpretative process while maintaining flexibility and scientific rigor, multiple collaborative discussions were held with senior researchers specializing in social work, which helped minimize the influence of personal assumptions.

### Ethical Considerations

This study received approval from the Swedish Ethical Review Authority (Dnr 2021-02790; 2023-05055), adhering to ethical principles such as those outlined in the Declaration of Helsinki (WMA, 2024). Throughout the research, ongoing discussions on self-reflexivity, ethical considerations, and power dynamics were held among the research team.

Grounded in the sociological theory of childhood (James et al., 1998; Mayall, 2000), this study views childhood as a social construct shaped by cultural, historical, and societal contexts. This perspective emphasizes researching with children, recognizing them as active agents with their own perspectives and experiences (Bodén, 2021). It challenges traditional views of children as passive “becomings,” instead highlighting their role as competent “beings” who contribute to their social worlds (Heimer et al., 2018). The theory also stresses the importance of incorporating children’s rights perspectives, requiring careful consideration of power, agency, and ethical responsibility when engaging with young participants (Källström, 2017). Thus, the study aims to treat adolescents as social and competent actors (Rasmusson et al., 2010; Toros, 2021) and knowledge carriers (Fricker, 2007), while ensuring their vulnerability does not define the relationship or hinder their contribution to the research.

In line with this approach, I maintained a neutral position by having no prior relationships with the adolescents or their parents. This neutrality was intended to foster open and honest dialogue during the interviews. To further support this, the youth interviews began with an informal and hopefully relaxed conversation, allowing us to get to know each other while acknowledging the complexity of discussing sensitive experiences with someone significantly older and unfamiliar. Also, due to previous negative care experiences, it was important to address overall trust issues concerning adults. Before starting the interviews, and in order for the adolescents not to feel inferior to the adult agenda, I emphasized that we were working together to examine participation from our diverse perspectives. Accordingly, emphasis was placed on the adolescents’ agency, allowing them to control their participation and feel empowered throughout the process. Although consent was not required from parents for adolescents over 15, efforts were made to ensure the adolescents understood they could withdraw or skip questions at any time. This turned out to be an important aspect as the majority of the young people said they appreciated this at the end of the interview, and that this affected their trust in me and facilitated an overall non-tokenistic participation in the study.

The interviews with parents were conducted differently from those with adolescents, as all parents preferred digital platforms, which were accommodated. Discussing psychiatric inpatient care often evoked strong emotions, as parents grappled with the emotional toll of their child’s enrollment and the challenge of navigating a fragmented health care system (cf. Merayo-Sereno et al., 2023). The digital format made it harder to detect emotional responses and provide immediate support during distress. However, my prior experience in CAP likely contributed to balancing empathy for their experiences with maintaining a productive interview atmosphere. Although parents initially voiced frustration and disappointment with the quality of care, the interviews gradually shifted to explore more nuanced aspects of participation during care episodes.

## Results

The results are presented as six interconnected themes, culminating in a main interpretation (see Table 1). A shared experience among respondents is that inpatient care operates within its own logic, often perceived as a struggle marked by power hierarchies and feelings of being an “imposter.” This uncertainty about whether their mental health problems justified care sometimes led to an exaggeration of symptoms. Previous care episodes were characterized by confusion and a lack of clarity about procedures, and the findings reflect these shared experiences of both young people and parents.

**Table 1.** Overview of Themes and Main Interpretation.

Main Theme	Sub-Themes	Key Insights
Care On Different Terms	-Loss of control and uncertainty in inpatient care	Adolescents feel excluded from decisions and experience guilt. PIBA offers freedom and reduces emergency unit stress.
	-Benefits of PIBA	
It is all about trust	-Trust between adolescents, parents, and staff	Trust is essential for adolescents to seek help. Negative past experiences undermine trust, affecting participation.
	-Impact of past care experiences	
Not having to fall apart to receive help	-Barriers to accessing care	Adolescents value not having to exaggerate symptoms for care access. PIBA offers validation without the need to “prove” their struggles.
	-The importance of being taken seriously	
Participation as holding on and letting go	-Balancing adolescent autonomy and parental involvement	Adolescents want guidance without losing control. Parents struggle between supporting and letting go during PIBA decisions.
A joint relief	-PIBA as supportive for both adolescents and parents	PIBA provides respite for adolescents and parents. It reduces pressure and promotes independence while offering safety nets.
	-Emotional distance in care	
Being addressed directly	-Importance of direct communication with adolescents	Adolescents feel respected when addressed directly. Engagement in contract discussions improves participation and fosters understanding.
	-Involvement in care decisions	
Main interpretation: Togetherness in inpatient care	-Trust and collaboration among adolescents, parents, and staff	Participation is a relational process built on trust, flexibility, and togetherness. Recognizing adolescents as knowledge carriers cultivates epistemic justice and promotes recovery. PIBA encourages co-created care involving all stakeholders.
	-Ecological and relational participation	

In this section, full quotes are utilized to illustrate key findings while also integrating shorter excerpts from participants’ statements directly into the descriptive text. This approach allows for a more fluid narrative while still grounding the analysis in the participants’ voices. The embedded excerpts highlight specific expressions or sentiments that support the interpretation without interrupting the flow of the results. Full quotes are used where a more comprehensive context is necessary to deepen the reader’s understanding.

### Care on Different Terms

Inpatient care often feels like a loss of control for young people, who feel excluded from decisions and guilty about “taking resources from others.” Initial experiences in CAP emergency units are often confusing, with unclear benchmarks leading to doubt about help availability. A young woman with PIBA experience found it generally positive, despite a confusing admission process that differed from expectations. During PIBA, she felt more involved in decisions, while finding reassurance in the contract’s facilitation of care access.

The best thing about PIBA is the freedom it provides. The discharge process feels less daunting because I know I can lean on the fact that I have the option to return without going through the emergency unit. Having a “backup” plan is really reassuring. It’s comforting to know I have the contract, but I don’t always need to use it to feel better. That’s the key function that PIBA serves. (Ester, adolescent)

Parents believe that increased participation through PIBA reduces the high-stress scenarios often seen at emergency units, fostering a “friendlier” and more “humane” care environment. PIBA is seen as surpassing traditional inpatient care, but parents emphasize the need for its theoretical promises to be realized in practice to rebuild trust. A parent with PIBA experience highlighted its more delicate and compassionate approach compared to compulsory care, emphasizing its positive impact on treating young people in crisis.

It felt much less harsh—like, it was almost a “strategy,” I guess. When she chose voluntary care, the treatment was softer. It wasn’t like being just another person forced into care and treated in a strict, tough manner. (Christoffer, parent)

### It Is All About Trust

Adolescents’ prior care experiences impact their trust in inpatient care, consequently affecting their openness to PIBA. They question seeking help, citing lack of trust as a major barrier: “How can they listen properly if they don’t trust what I say or think I have my own agenda?”. This mistrust hinders building meaningful relationships with staff. Re-establishing trust requires mutual confidence—adolescents must believe in staff’s support capabilities, while staff must trust adolescents’ self-understanding.

I think it’s based on … it’s probably down to how much faith you have in the care you’ll receive that affects whether you would dare to self-admit or not. And I don’t think I trust CAP to the extent that I would have wanted to enroll myself. It requires that they (the staff) know what they’re doing. (Olivia, adolescent)

Rebuilding trust and repairing broken relationships are paramount for young people to consider inpatient care. Trust between them and staff is described as crucial for effective care and influences whether seeking support or not. Many feel traditional inpatient care fosters distrust, with staff assuming the worst: “There was never any trust … and then you can’t get any help.” As trust diminishes, so does hope in inpatient care as a supportive environment, leaving adolescents guarded when accessing services.

To be able to trust someone to sort of … take care of something valuable, they (staff) have to be able to make sure that they don’t lose valuable experiences from previous situations (enrollments). If someone has the habit of … well … “stealing” from your wallet when they get to hold your bag, then you wouldn’t trust them to give them your bag the next time. Because you don’t know if they’ll steal from your wallet again, or take something else from it. (Mika, adolescent)

At the same time, parents talk about trust as a prerequisite for both accepting PIBA and daring to leave their child in inpatient care. When trust is present, it brings a sense of calm, and when it is absent, you are left powerless. Trust is something that is described as occurring in the personal meeting with staff, which means that the relational aspect between young people, parents, and staff is fundamental for trust to be achieved.

In order to feel that it (PIBA) is helpful for me as a parent … I think it boils down to … how she is treated by the CAP doctors in general. And that we as parents are seen and heard in such a way that makes us feel at ease with the PIBA terms. (William, parent)

### Not Having to Fall Apart to Receive Help

Accessing inpatient care is often difficult, with adolescents encountering resistance and uncertainty from staff. This may stem from limited resources, strict criteria, and challenges in assessing complex mental health needs. The CAP emergency unit is described as high-tension, with exhausting screening processes. According to young people, a key benefit of PIBA is the opportunity to bypass the emergency unit, be taken seriously, and avoid having to “convince” CAP of their need for help.

It (PIBA) means I don’t have to prove to anyone that I “deserve” this help. I don’t have to “compete” with other young people. So, yes, the idea of not having to prove anything in the way I needed to (to be enrolled in traditional inpatient care), that would be really helpful. (Jacob, adolescent)

The struggle often lies in wanting to be heard but not feeling genuinely listened to, in a context where discussions happen about, rather than with, adolescents. As a young person, you want “people to be honest about what’s happening and not to be treated like a naive child.” However, in moments of vulnerability, it can be difficult to reach adults, especially when the adults themselves are unaware of this or do not act in ways that make it easier for young people to express their needs. During the interview, the adolescent Ester captures this sentiment by saying:

But sometimes it feels like the doctors don’t want to listen to you.

How can you tell?

You can tell because … they focus more on talking to the parents. Doctors who genuinely listen to the patient don’t prioritize speaking with the parents. If I meet a doctor who insists on waiting until my parents arrive, or if the meeting with my parents lasts longer than with me—these are red flags that it won’t go well. It’ll be based on what my parents think and feel.

Parents speak in similar terms, that is, they emphasize the importance of being able to decide and define their own needs during an admission procedure. Rather than presenting a full history of suffering, PIBA is described as facilitating the admission process while ensuring that the young person is taken seriously. Parents generally describe this as care-saving and dignified.

I think PIBA would have been really helpful in that situation, because then she wouldn’t have to doubt whether she’ll get help. It would give her the confidence to say, “Okay, I can decide for myself when I’m struggling and at risk of hurting myself.” Knowing she could go there and be allowed to stay would make a big difference. For her to feel, “I know what I need, and I can make that decision,” I think would have been very helpful. (Iris, parent)

### Participation as Holding on and Letting Go

None of the adolescents wish to decide on PIBA enrollment alone. They value parental involvement, seeing it as useful when it does not dominate decision-making. Parents are viewed as “guides” helping navigate inpatient care decisions while building confidence to seek support. Rather than complete independence, young people emphasize dialogue to foster their willingness and ability to participate in care.

If I was to use PIBA, I don’t think I’d call the ward myself. Someone would have to do it on my behalf. Yeah, I think it’s better that someone does it for me. (Gabriel, adolescent)

A similar argument is made by Mika, who sees the benefit of discussing a potential admission with an adult whom he trusts. Exploring together what could be a possible solution is described in positive terms, where you can understand it as the young person first taking a step forward and then a step back while waiting for a response from a parent.

I would like to have support, asking my parents “What do you think? I think like this.” I’d like to have someone to sort of reason with. At the same time, I don’t want them (parents) to make the decision for me or say “no, you’re not ‘sick’ enough to get help.” But I want to be able to say “okay, now I’m going to get help.” But I would like to have someone to talk to … to say “I don't know what to do right now … what do you think?” (Mika, adolescent)

Parents emphasize that participation should involve increasing the young person’s responsibility while they, as parents, step back—a process that can feel unfamiliar and intimidating. However, none believe the decision to use PIBA should be managed entirely by the young person. Instead, parents see themselves as implicitly part of a process aimed at fostering future independence.

I think I would be proud if he takes responsibility for noticing that “now it’s (the mental health situation) going in the wrong direction.” I would think that was good, and it’s also a step towards independence in a way. Because when he gets older, he might not want to involve me too much, so it’s natural that he wants to manage better on his own. (Agnes, parent)

Parents believe young people need support navigating life due to poor mental health, even as they take on more health care decisions. This requires balancing child-centered and family-centered care, alternating participation and protection. The evolving parental role involves a shift from providing fundamental security to fostering the child’s growing decision-making ability with age, which is important for staff to understand.

When her mental health is worse … as it is at the moment, she sleeps in my bed because she’s afraid that she’ll hurt herself at night. So, I think that, for better or for worse, I’m a very important part of her basic security when she deteriorates. If she gets worse and this (PIBA) were an option, then I would have to help her. So even if she were to make a decision and it’s of her own choosing, I think I would still have to guide her. (William, parent)

Inpatient care is described as hierarchical, where previous experiences and relationships with staff—especially doctors—influence whether parents dare to let go or take a more controlling role to ensure their child receives promised care. These hierarchies ultimately affect the degree of participation exercised in inpatient care.

Participation means having a clear plan, being informed about decisions, and having them explained, along with how to achieve specific goals. However, during our inpatient care experience, this was lacking. It shouldn’t be that hard to explain things to me as a parent in an informative and understandable way. Instead, it felt like the doctors excluded us, avoiding involvement with us entirely. (Christoffer, parent)

### A Joint Relief

Adolescents view hypothetical access to PIBA as “supportive” and “reassuring,” promoting integrity and self-determination in inpatient care. It offers a sense of respite and helps prevent harmful behaviors without feeling like an “imposter.” While participation in care is valued, they acknowledge the need for the care system to prioritize safety and protection when necessary. Temporarily relinquishing participation for stability is seen as acceptable but does not mean being excluded from involvement altogether.

The healthcare system must retain enough authority to ensure effective care. For example, if I just need to stay in bed to avoid self-harm, I don’t need much input on how staff prevent it. However, I want to participate in decisions unrelated to self-destructive behavior, like whether I should attend school and how many days I should go. (Olivia, adolescent)

Adolescents believe deciding on inpatient care access and admission without parents can improve mental health. This distance allows authentic expression of feelings without concern for others’ reactions. In traditional care, constant parental presence can feel restrictive, whereas self-admission seems to better address their need for relief from managing parental emotions.

Many adolescents are forced to have their parents with them on the ward, even if they don’t want them there. Staff claim “they have the right to be here because they’re your guardians,” leaving no option to send them away. As a result, many young people pretend to be okay. No 16-17-year-old spends that much time with their parents, and it’s difficult to be honest about how you truly feel when your mom is sitting right there. As you get older, you try to gain independence, but staff “glue” you back together, forcing constant 24/7 closeness. (Ester, adolescent)

Parents view PIBA as a “relieving” and “unique” asset, regardless of its use. This care form is valued for benefiting both young people and parents by providing increased access to inpatient care compared to traditional admissions. Being offered the intervention makes parents feel that “at least something is happening in a positive direction, and you take what little there is.” In essence, there is an opportunity to rest from the immediate responsibility for the child’s health.

There were days when I was incredibly tired, and I thought, “It would’ve been helpful if she could have attended a day program” and had her meals taken care of by someone else, allowing me to focus on resting during the day. This kind of admission (PIBA) would have been invaluable. (William, parent)

Parents describe inpatient care as stressful and challenging, often leaving them feeling inadequate in balancing family needs. PIBA, not requiring parents to stay on the ward, is seen as beneficial, allowing focus on work and other children at home. Reflecting on past experiences, parents feel staff often lack empathy and fail to grasp the family’s collective trauma. Ingrid shares her perspective on what she believed PIBA would mean for her daughter.

It would be a huge relief, as we also have to care for our younger daughter at home. The healthcare system often assumes parents have only one child, but we have two, and we also have to manage life outside of inpatient care.

So, if I’m understanding you correctly, PIBA could provide some relief for you as a parent?

Yes, that’s right.

### Being Addressed Directly

To optimize PIBA, it is said to be important to actively involve the young person, with staff engaging in dialogue with them directly, not just their parents. This approach significantly impacts how adolescents perceive the PIBA offer. The contract signing should reflect the young person’s needs and questions, rather than being a conversation solely between CAP and parents. Adolescent Ester describes how this process prioritized her involvement.

I had support from my care worker, so I felt safe, even though I usually find large meetings challenging, where everyone’s focus is on me, and I feel like people are talking about me, not to me. When I was younger, staff didn’t speak to me directly but instead talked about me to my parents in front of me—I felt like I was just there for show.

But this meeting was different?

Yes, because she made sure everyone spoke directly to me, which made me feel more comfortable. The meeting wasn’t long, and I wasn’t overwhelmed with new information. I could easily understand what the situation would involve from the start.

Parents stress the importance of signing the PIBA contract under youth-adapted conditions. The process benefits from dialogue and active youth involvement, making it “flexible and person-centered.” Christoffer, a father with practical experience emphasizes that adolescents must fully understand the contract’s implications—for PIBA to effectively support mental health, the contract must be clear and accessible.

We were involved in the design of the contract, and we supported her (the daughter) throughout the whole process. Because we wanted to be familiar with what it was about and make sure that the contract was designed in such a way that she would appreciate it, and not just sign something for our sake.

For participation in inpatient care to be effective, the contract should be flexible, enabling the young person to recognize that they have clear support when difficulties arise. Parents stress the importance of dialogue between the young person and the staff member offering PIBA—whether in outpatient or inpatient care—as a key element in fostering meaningful participation.

If she (the daughter) feels pressured or cornered, she reverts to basic behaviors, and in the worst case, suicidal thoughts. I see participation as challenging for a child dealing with poor mental health. She needs to feel genuinely involved to avoid being overwhelmed during the contract discussion. It’s crucial that she’s fully ‘on board’ with it. (William, parent)

### Main Interpretation: Togetherness in Inpatient Care

Young people and parents express a lack of trust in inpatient care based on prior experiences, which complicates their willingness to consider PIBA. However, PIBA’s potential as a “different” form of care—focused on promoting treatment, reducing suffering, and granting individuals the ability to oversee their care—emerges as instrumental. Viewing young people as epistemic agents allows them to define their own needs without having to convince the system of their worthiness for care (Fricker, 2007). This approach fosters a sense of control, contrasting with the uncertainty and stress commonly associated with traditional care settings. PIBA’s success relies on involving the young person’s ecological context (Bronfenbrenner, 1979) and ensuring that staff treat the family in a youth-friendly and participatory manner. Recognizing adolescents as capable actors during the contractual process appears to promote participation and recovery-oriented care.

Central to this dynamic is the significance of relationships in fostering participation during inpatient enrollment. Participation does not occur automatically with PIBA but develops through ecological interactions between young people, parents, and staff (Bronfenbrenner, 1979). For adolescents, participation is shaped by self-perception, their relationships with parents, and the supportive role of staff. When adolescents feel compelled to exaggerate symptoms to access care, it creates a sense of imposture that undermines their sense of participation and reduces trust in the care system. Conversely, trust in parents and staff to validate their need for care facilitates a less conditional and anxiety-driven admission process.

To promote participation and epistemic justice in inpatient care, parents and staff are key in cultivating a climate of togetherness while respecting young people’s subjective knowledge (cf. Fricker, 2007). Participation balances care dependence and decision-making autonomy as adolescents transition to adulthood. Flexibility within ecological systems seems essential for this balance, as opposed to rigid systems and hierarchical knowledge structures that risk disrupting co-creative relationships and undermining youth participation.

## Discussion

This study sought to explore adolescents’ and parents’ attitudes toward youth participation during PIBA enrollment in psychiatric inpatient care, and its role in recovery from mental health problems. Through semi-structured interviews, it provides in-depth insights into the significance of advancing participation in CAP settings, bridging theoretical discourse with practical experiences and outcomes.

The main findings indicate that participation is a *joint process* that involves trusting relationships fostered by togetherness and flexibility. Furthermore, because CAP is an organization characterized by hierarchies of knowledge, it seems important for adults to legitimize young people’s ability to define their individual care needs rather than dismissing them, which may result in tokenistic participation and pose organizational barriers to meaningful engagement. Thus, for participation to become more than policy and translate into effective practice, power and knowledge need to be re-negotiated between adolescents, parents, and staff. Additionally, given the crucial role of staff in implementing young people’s participation, it is essential for them to reflect on their own epistemic beliefs, as these are likely to influence the practical outcomes of inpatient care.

Young people without a PIBA contract believe that easier access to inpatient care, without the need to fight for it (cf. Hultman & Hultman, 2023; Värnå et al., 2025; Wallström et al., 2021), could enhance participation. However, their previous negative experiences, characterized by distrust and a lack of “youth-friendly” care (cf. McCann & Lubman, 2012; Moberg et al., 2022), make them uninterested in being offered such contracts. These strained relationships undermine trust in and connection with staff and the care system, hindering participation and complicating relationship-building (Ballesteros-Urpi et al., 2019; Hense & McFerran, 2017). From the perspective of epistemic injustice (Fricker, 2007), these dynamics highlight testimonial injustice, where young people’s accounts are dismissed or undervalued due to systemic biases. Distrust and disconnection further limit young people’s ability to be recognized as epistemic subjects, as their perspectives are often overshadowed by staff’s authority within hierarchical knowledge systems (cf. Fricker, 2007; Moberg & Schön, 2022).

Similarly, hermeneutical injustice occurs when adolescents struggle to articulate their mental health needs due to a lack of shared language or frameworks, often in care settings where adult perspectives dominate (cf. Carel & Györffy, 2014; Greenblatt et al., 2024). This reinforces a cycle of exclusion, where their voices remain unheard and they become further alienated from care processes (cf. Newbigging & Ridley, 2018). Although the concept of epistemic injustice highlights how young people’s accounts may be dismissed it is important to recognize that this dynamic is rarely a matter of outright disbelief. Rather, it reflects a complex interplay between staff perceptions and structural constraints within the health care system. For instance, while staff may acknowledge adolescents’ self-reports, institutional barriers—such as strict admission criteria or limited resources—may prevent them from acting on these accounts. This tension can inadvertently lead to young people feeling unheard or invalidated, despite staff’s intentions, which is why attentiveness to adolescents’ needs is essential in aligning mental health care (Nakash et al., 2018). Moreover, in order to cultivate genuine participation and counteract epistemic injustice, care should value young people’s knowledge and experiences while ensuring that participation is non-tokenistic (cf. Daly, 2018; Lundy, 2007; Värnå et al., 2025). This requires creating environments where adolescents’ voices not only receive recognition but actively shape care decisions, thereby challenging the power hierarchies that contribute to their silencing.

On a hypothetical level, young people believe that the increased participation that PIBA offers may prevent mental health problems and reduce self-destructive behavior, but without trusted support, being “abandoned” in inpatient care can hinder recovery. PIBA involves a gradual transfer of mental health management from parents to children (cf. Giarelli et al., 2008). Despite this shift, parents are often crucial in fostering participation and aiding recovery (Bjønness et al., 2022; Moberg & Schön, 2022). Including parents during enrollment to discuss the pros and cons of inpatient care is essential, as studies emphasize the need to define parental roles in recovery (Moberg, 2024; Moberg et al., 2022).

While epistemic injustice highlights how youth voices are overlooked, ecological systems theory provides a broader framework for understanding how participation is influenced by multiple layers of social and institutional structures. Applying the lens of Bronfenbrenner (1979) reveals how these dynamics are embedded within multiple, interconnected systems. At the microsystem level, the immediate relationships between adolescents, parents, and staff shape participation. Strained trust at this level can hinder engagement, while supportive, communicative relationships can empower young people (Rayner et al., 2018). The mesosystem, encompassing the connections between these microsystems, influences how well participation structures like PIBA function. For instance, when parents and staff collaborate effectively, adolescents are more likely to feel supported and included. Further, at the exosystem level, institutional policies and systemic barriers—such as rigid care protocols—affect the accessibility and effectiveness of participation opportunities. The macrosystem, reflecting broader societal attitudes toward youth autonomy and mental health, further shapes how adolescents’ voices are perceived and valued within care settings (Yamaguchi et al., 2023). From this perspective, adopting an ecological framework that integrates participatory structures seems essential for CAP to deliver recovery-oriented care (Moberg & Schön, 2022; cf. Bronfenbrenner, 1979). As such, this approach reframes parents as key allies in supporting youth participation (Moberg, 2024; cf. Bjønness et al., 2022).

Scholars describe adolescence as a transitional period marked by a delicate balance between dependence and the pursuit of independence (cf. Bjønness et al., 2020). As regards PIBA, it reflects the “yoyo transitions” described in research, where non-linear shifts between participation and reliance on others are evident (Biggart & Walther, 2006; Skogens et al., 2017; Spånberger Weitz, 2011). In psychiatric inpatient care, these transitions present challenges for staff, who need to balance young people’s rights to both participation and protection in vulnerable situations (cf. Coyne & Harder, 2011; Lundy, 2007; Moberg, 2024). While transitions between youth dependency and adult independence (Spånberger Weitz, 2011) are natural, they can be complicated by mental health problems. By fostering trust and respect, PIBA has the potential to support these developmental processes, promoting both autonomy and responsibility in managing mental health.

By merging epistemic injustice with ecological systems theory, this approach provides a holistic perspective on the complex barriers adolescents face. For example, testimonial injustice is often reinforced by mesosystem dynamics where staff may prioritize parental input over adolescents’ voices, reflecting deeper macrosystemic biases about youth credibility. Similarly, hermeneutical injustice is exacerbated by exosystem factors—such as rigid institutional protocols—that fail to accommodate adolescents’ communicative needs, limiting their capacity to participate fully.

### Strengths and Limitations

One of the study’s strengths is that it combines the perspectives of both young people and parents regarding youth participation, and may therefore contribute to an increased understanding of how participation contribute to recovery from mental health problems. However, methodological considerations also need to be addressed when interpreting the results.

First, only two out of 10 respondents have practical experiences of PIBA, pointing to the fact that the majority of the material presented here consists of hypothetical assumptions about the intervention and its relevance for participation in inpatient care (cf. Meskus & Tikka, 2024). Although this study does not aim to evaluate PIBA specifically, including more participants with experience of PIBA could have provided deeper insights into the practical implementation of participation work, rather than limiting the discussion to a more hypothetical level. This must be considered as one of the study’s most significant limitations.

Second, individual interviews were conducted due to the sensitive nature of the subject matter at hand—that is, sharing experiences of being in inpatient care. This setting does not offer the opportunity for participants to build on each other’s ideas or provide peer support (Hassler et al., 2024). It may thus be beneficial to harness knowledge about participation through, for example, focus group interviews. In such a setting, participants are able to support each other in order to reduce the unavoidable power disparity that occurs in an interview setting, regardless of how diligently the interviewer attempts to manage this.

Third, my experience of having worked in CAP has both advantages and disadvantages in the context of this study. On the one hand, it has provided me with access to the target group and the ability to discuss potentially sensitive topics. On the other hand, it has also introduced a risk of compromising the objectivity required to interpret the material in an impartial manner. Considering the contrast between actively participating in a practice and observing it from the outside emphasized the significance of maintaining reflexivity throughout the research process (cf. Berger, 2015; Olmos-Vega et al., 2022). However, in terms of reflexivity, this has been addressed through regular discussions with senior researchers who have also reviewed manuscript drafts and provided feedback on the analysis process.

## Implications for Practice and Future Directions

The findings show that although staff in CAP often demonstrate a positive attitude toward participation (cf. Moberg & Schön, 2022), young people and parents frequently encounter obstacles in the inpatient care setting. This environment involves a constant tension between promoting the participation of young people and ensuring their protection (cf. Moberg, 2024). In practice, this duality tends to favor protection, making participation challenging to implement for adolescents with extensive care needs. The study highlights this balancing act while offering insights into optimizing participation in inpatient care. The results indicate that youth participation in inpatient care is not static but dynamic, evolving in frequency and intensity through interactions with adults. Adolescents, regardless of their mental health status, should be acknowledged as individuals approaching adulthood, learning to take on greater participation in their everyday lives.

To prevent epistemic injustice and reduce barriers to participation, parents and staff would likely benefit from viewing these developmental transitions as integral to the participatory process. Accordingly, in order to strengthen epistemic justice among young people in vulnerable situations and to avoid further distrust and dislocation (Edwards et al., 2015; Lantto et al., 2023; Lindkvist et al., 2022), CAP should therefore acknowledge participation as a joint effort among the triad consisting of adolescents, parents, and staff (cf. Bronfenbrenner, 1979; Wiklud Gustin, 2021). Developing CAP into a recovery-oriented system thus requires shifting from “moments of participation to a participatory culture” (Bromark, 2024, p. 52), while emphasizing the importance of experience-based knowledge as a cornerstone of care.

Although this study provides a comprehensive understanding of how participation is experienced in inpatient care, further research is needed. This research should examine specific strategies for strengthening youth participation in psychiatric inpatient care, particularly through interventions like PIBA. To be more precise, further research is needed to explore the extent to which PIBA genuinely supports autonomy, particularly in balancing youth decision-making with parental and institutional involvement. Further, exploring the role of “imposter syndrome” in inpatient care settings and its implications for mental health (Barari et al., 2024) could provide deeper insight into how participation and recovery are facilitated—or hindered—within this context.

## Supplemental Material

Supplemental Material - “The Idea of Not Having to Prove Anything in the Way I Needed, That Would be Really Helpful”: Exploring Adolescents’ and Parents’ Understanding of Youth Participation During Patient-Initiated Brief Admission Enrollment in Psychiatric Inpatient Care Supplemental Material for “The Idea of Not Having to Prove Anything in the Way I Needed, That Would be Really Helpful”: Exploring Adolescents’ and Parents’ Understanding of Youth Participation During Patient-Initiated Brief Admission Enrollment in Psychiatric Inpatient Care by Jennie Moberg in Qualitative Health Research

## Notes

1. For clarity, the terms “adolescents” and “young people” are used synonymously in this study to encompass individuals within this developmental stage. Similarly, “professionals” and “staff” are used interchangeably to refer to all individuals providing care within the inpatient setting.

2. An English translation of the full interview guide is available in the online supplemental material.
