# Supplementary material for: “The Idea of Not Having to Prove Anything in the Way I Needed, That Would Be Really Helpful”: Exploring Adolescents’ and Parents’ Understanding of Youth Participation During Patient-Initiated Brief Admission Enrollment in Psychiatric Inpatient Care
Source: Qual Health Res. 2025 Jun 7;36(13):1445–61. doi: 10.1177/10497323251340935 (PMC13672931; doi:10.1177/10497323251340935)
Supplement: Supplementary file 1 [file sj-pdf-1-qhr-10.1177_10497323251340935.pdf]

*Background information*

-Gender (girl/boy/other)? Age? Ethnicity? Family structure? School/job/other activities?

-How long have you had contact with CAP? When was your most recent admission?

-What does your current care situation look like?

*Previous experiences with psychiatric inpatient care*

-Can you tell me what happened the last time you were admitted? Follow-up questions if needed (Were your parents involved? Type of care? Treatment approach? Degree of participation)?

-Looking back, was inpatient care designed in a way that suited you? Was there anything you wished for that wasn't available? If not, what is needed for care to support you?

*Participation*

-Today, psychiatry emphasizes giving patients participation in their care—patients should have influence, access to information, and the ability to make decisions. Is this something you've heard about?

-What does participation in inpatient care mean to you?

-Were you able to make any decisions during your admission? If not, what would you have liked to decide on? What is important to you during inpatient care?

-Was there a situation where you couldn't or weren't allowed to make decisions?

-Are there situations where you didn't want to make decisions? Are there things you feel it's better for others to decide on in inpatient care? Can you describe such a situation?

-Have your parents influenced anything when you needed inpatient care (e.g., care content, desired treatment approach, contact person)?

*Information about PIBA*

Introduce the care model. Explain that there's a specific contract where the young person, parents, and staff determine the content together.

-Have you heard of PIBA?

-If you had the opportunity to decide for yourself when to be admitted, do you think it would make a difference? How? How would it feel to have such a contract?

-If you had access to this care model, would you want support in the decision-making process for admission? Who would you want support from, and in what way (e.g., calling the ward to check for availability, help with transportation)?

-What would you like to see included in the contract?

-What would be important for you to feel comfortable using PIBA? Would anything specific be required of you to manage it?

-Reflecting on previous admissions, do you see any risks in having increased participation when you're feeling mentally unwell?

*If the young person has access to and/or has used PIBA*

-How long have you had access to PIBA? Have you used it so far?

-Would you like to share how the process unfolded for you to gain access to PIBA? (What information were you given? Were you involved in planning the setup? What was the dialogue like between you, your parents, and the staff?)

-What significance has PIBA had for you? Has access to PIBA affected your schooling, family relationships, friendships, etc.?

-What do you think is required of you to handle PIBA effectively?

-If you've used PIBA, were you the one who decided to go to the ward? Would you like to share how that decision-making process worked?

-Have there been any conflicts between you and staff/parents during the care period? Can you describe what these conflicts were about?

*Follow-up questions if not already addressed*

-When you've been admitted, has the relationship with the staff been important to you? What do you think staff should consider to enable you to make more decisions in inpatient care?

-What do you think has influenced how staff reasoned or decided about your ability to make decisions during admission?

*Other/ending the interview*

-Do you think I've forgotten to ask anything important, or is there anything you'd like to add that you think I should know?

*Background information*

-Mother/father/guardian? Age? Ethnicity? Occupation?

-How long has the family been in contact with CAP? When was the most recent admission?

-What is your child/children's current (care) situation?

*Previous experiences with psychiatric inpatient care*

-Can you describe how the process unfolded when your child/children were admitted? (Who was involved, type of care, treatment approach, degree of participation, etc.)?

-Looking back, was the inpatient care structured in a way that suited you and your child/children? Was there anything you wished for that wasn't provided?

-What impact do you think the possibility of inpatient care has had on your child/children's well-being?

*Participation*

Today, psychiatric care emphasizes involving patients and their families in the care process. This means patients should have influence, access to information, and the ability to participate in decision-making.

-When you reflect on it, what does participation in inpatient care mean to you?

-Were you and your child/children allowed to make decisions during admission? If not, what would you have wanted to decide?

-Were there situations where you and your child/children wanted different things? What happened then?

-Are there things you felt it was better for others to decide? Can you describe such a situation?

-Do you, as a parent, develop or need specific strategies to ensure participation in inpatient care?

-Have you influenced the care provided when your child/children needed inpatient care (e.g., treatment content, preferred approach, key contact person, etc.)?

-What is important to you as a parent when accompanying your child/children in inpatient care?

*Information about PIBA*

Introduce the care model. Explain the specific contract, where the young person, parents, and staff jointly determine the content.

-What would be required for you as a parent to consent to such a care model?

-If your child/children had the opportunity to decide independently when they want to be admitted, do you think it would make a difference for your situation? In what way?

-How do you think it would feel for you as a parent if your child/children had such a contract?

-How do you imagine the decision-making process about using PIBA would look in your family, and how could the young person be supported in such a process (e.g., discussing admission with the child, calling the ward to check for availability, helping with transportation)?

-What would you want included in the contract? What is important for you as a parent to ensure during a PIBA admission?

-What would be important to you to allow your child/children to use a care model like PIBA? Would anything specific be needed for your family to manage it?

-Reflecting on previous admissions, are there risks for your child/children in being given increased participation when they are feeling mentally unwell?

*If the parent has experience with PIBA*

-How long has your child/children had access to PIBA? Have they used it so far?

-Would you like to share how the process unfolded leading up to your child/children receiving PIBA? (What information was provided to the

family, were you involved in planning the PIBA setup, what was the dialogue like between your child/children, yourself, and the staff?)

-What significance has PIBA had for you as a parent?

-What would you say is required of your child/children to handle PIBA effectively?

-If your child/children have used PIBA, how has the admission process worked?

-Have there been any conflicts between you, your child/children, and the staff during the care period? Can you describe what these conflicts were about?

*Follow-up questions if not already addressed*

-When you have accompanied your child/children in inpatient care, has the relationship with the staff been important to you? What do you think staff should consider to enable a child to have more decision-making power during inpatient care?

-What do you think is important for inpatient care staff to consider in their interactions with you as a parent?

-Have there been any (specific) challenges in inpatient care that you have noticed or reflected on during your stay?

-What do you think has influenced how the staff have reasoned or decided about your child/children's ability to make decisions during admission?
